# Global Proteomics Analysis of Lysophosphatidic Acid Signaling in PC-3 Human Prostate Cancer Cells: Role of CCN1

**DOI:** 10.3390/ijms25042067

**Published:** 2024-02-08

**Authors:** Pravita Balijepalli, Guihua Yue, Bhagwat Prasad, Kathryn E. Meier

**Affiliations:** Department of Pharmaceutical Sciences, College of Pharmacy and Pharmaceutical Sciences, Washington State University, Spokane, WA 99202, USA; pravita.balijepalli@wsu.edu (P.B.); guihua.yue@wsu.edu (G.Y.); bhagwat.prasad@wsu.edu (B.P.)

**Keywords:** lipid mediators, prostate cancer, matricellular proteins, extracellular matrix

## Abstract

Cysteine-rich angiogenic factor 61 (CCN1/Cyr61) is a matricellular protein that is induced and secreted in response to growth factors. Our previous work showed that 18:1-lysophosphatidic acid (LPA), which activates the G protein-coupled receptor LPAR1, induces CCN1 between 2–4 h in PC-3 human prostate cancer cells in a manner than enhances cell-substrate adhesion. While the time course of induction suggests that CCN1 contributes to intermediate events in LPA action, the roles of CCN1 in LPA-mediated signal transduction have not been fully elucidated. This study utilized a comprehensive global proteomics approach to identify proteins up- or down-regulated in response to treatment of PC-3 cells with LPA for three hours, during the time of peak CCN1 levels. In addition, the effects of siRNA-mediated CCN1 knockdown on LPA responses were analyzed. The results show that, in addition to CCN1, LPA increased the levels of multiple proteins. Proteins up-regulated by LPA included metastasis-associated in colon cancer protein 1 (MACC1) and thrombospondin-1 (TSP1/THBS1); both MACC1 and TSP1 regulated cancer cell adhesion and motility. LPA down-regulated thioredoxin interacting protein (TXNIP). CCN1 knockdown suppressed the LPA-induced up-regulation of 30 proteins; these included MACC1 and TSP1, as confirmed by immunoblotting. Gene ontology and STRING analyses revealed multiple pathways impacted by LPA and CCN1. These results indicate that CCN1 contributes to LPA signaling cascades that occur during the intermediate phase after the initial stimulus. The study provides a rationale for the development of interventions to disrupt the LPA-CCN1 axis.

## 1. Introduction

Lysophosphatidic acid (LPA) refers to a family of simple lipids that are derived from membrane phospholipids. LPA acts as an agonist for G protein-coupled receptors; seven LPA receptors (LPARs) have been identified, and several are expressed in human prostate cancer (CaP) cell lines [1]. Our previous studies have shown that LPA is produced by human prostate cancer cells, where LPA can act as an autocrine mediator [2]. Oleoyl (18:1)-LPA can induce cell proliferation, migration, and adhesion in CaP cells, responses that are predominantly mediated by LPAR1 [1,2,3,4,5]. In terms of early events in signal transduction, LPA activates Erk MAPK, Akt, and FAK in CaP cells within a few minutes [1,5]. Other rapid responses characteristic of LPAR activation include increases in intracellular calcium and activation of Rho [6]. However, for LPA to initiate longer-term responses such as adhesion and proliferation, it is important to consider intermediate events that occur subsequent to the initial acute changes in signal transduction, linking early events in signaling to later changes in cellular function.

Among its many cellular actions, LPA has been shown to regulate proteins in extracellular matrix (ECM) [7,8,9]. LPA has been shown to induce expression of certain ECM proteins. One of these proteins is the matricellular protein CCN1, also known as Cyr61, which is induced in a Rho-dependent manner [10]. CCNs are secreted proteins that possess domains that allow them to interact with other proteins on the cell membrane (e.g., integrins) as well as with proteins in the ECM (e.g., fibronectin). In this way, CCNs can exert agonist-like activity and facilitate interactions between integrins and ECM proteins. The CCN family consists of six members; CCN1 and CCN2 are unique in that they are inducible. CCN1 can be rapidly induced, at the transcriptional level, by growth factors and by the phospholipid mediators LPA and sphingosine-1-phosphate (S1P) [11]. The induced protein is detected within a few hours after the stimulus, making CCN1 a potential mediator of subsequent signaling events. For example, Lau and colleagues demonstrated roles for CCN1 role in cell attachment, spreading, and chemotaxis in fibroblasts [12,13].

Not surprisingly, CCN1 is involved in cancer progression. Previous studies showed that LPA induces CCN1 in stromal and epithelial prostatic cells and that CCN1 is a viable marker for benign prostatic hyperplasia [14]. It has also been shown that LPA can activate Rho and induce CCN1 in astrocytoma cell lines [15]. CCN1 is induced through the activation of the transcriptional co-activator YAP in cancer cells [16]. Induced CCN1 is rapidly secreted from cells, appearing in the extracellular matrix [17]. Since CCN1 is a ligand for integrins, it is likely that further downstream signaling is mediated, at least in part, through integrins [18,19].

Our studies have shown that CCN1 is detected in human prostate cancer cells 2–6 h after the addition of LPA [5,17]. This led us to hypothesize that CCN1 plays a role in the intermediate phase of LPA response in these cells. Our previous results show that, in PC-3 cells, CCN1 plays an important role in LPA-induced cell adhesion that is observed after two hours [17]. Others have shown that CCN1 is required for PC-3 cell proliferation [20]. However, the full spectrum of LPA responses during an “intermediate” phase after LPAR1 activation has not been previously characterized. Also, a comprehensive analysis of the effects of CCN1 knockdown has not previously been reported. Global proteomics is an unbiased approach that can be used to quantify differences in protein expression between biological samples. The current study utilized global proteomics to address two goals. The first goal was to characterize changes in protein expression occurring three hours after LPAR1 activation in PC-3 cells. The second goal was to determine whether CCN1 is involved in any of these intermediate LPA responses. To address these goals, we performed global proteomics analyses comparing cells incubated with and without LPA, in the absence and presence of CCN1 knockdown.

## 2. Results

### 2.1. Identification of Proteins Differentially Expressed after LPA Treatment

**Figure 1:**
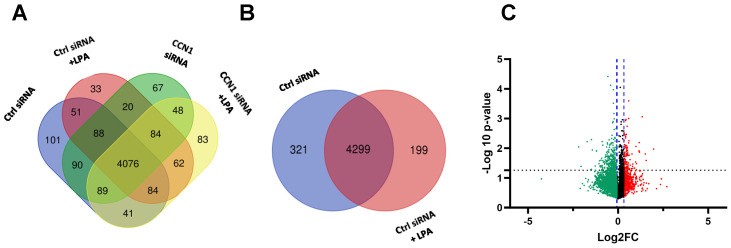
Proteins differentially expressed in PC-3 cells treated with LPA. (**A**) Venn diagram, prepared using Draw Venn Diagram (ugent.be), showing number of proteins detected in all four treatment groups used in this study (± LPA (3 h), ± control siRNA or CCN1 siRNA). (**B**) Venn diagram showing number of proteins detected in the two treatment groups treated with control siRNA, with and without LPA. (**C**) Volcano plot showing proteins (individual dots) differentially expressed between cells treated with and without LPA. FC = fold change (treated vs. untreated). The x-axis displays the fold-change; the y-axis shows statistical significance. The black dotted line indicates the threshold for statistical significance (*p* < 0.05). The blue dotted lines indicate the region without statistically significant fold-change. Red and green colors indicate proteins up-regulated and down-regulated, respectively, in response to LPA.

The first analysis examined differences in expression between untreated cells and LPA-treated cells. For both conditions, cells were incubated with a scrambled siRNA as a negative control for concomitant CCN1 siRNA treatments of other cell groups (see Section 2.2). Comparing cells incubated with and without LPA, untargeted global proteomics revealed 4299 proteins that were expressed in both treatment groups. There were 199 proteins uniquely expressed in LPA-treated PC-3 cells, compared to 321 proteins that were unique to the control group (Figure 1A).

Corroborating previous findings that LPA induces proteins that directly or indirectly modulate downstream signaling pathways, our analysis revealed that 256 proteins were up-regulated in PC-3 cells treated with LPA (Figure 1C). Notably, CCN1 was one of the most highly up-regulated proteins in the LPA treatment group. This is in agreement with our previous results demonstrating prominent induction of CCN1 by LPA in PC-3 cells [17]. The 3 h time point used for the LPA incubation was within the peak time of CCN1 induction and was approximately 1 h after maximal induction as previously detected by immunoblotting [17]. Among the top 20 proteins significantly up-regulated by LPA were metastasis associated in colon cancer protein 1 (MACC1), thrombospondin-1 (TSP1), transcription factor Jun-B, and Dickkopf-related protein 1 (DKK1). The most highly up-regulated proteins are listed in Table 1.

**Figure 2:**
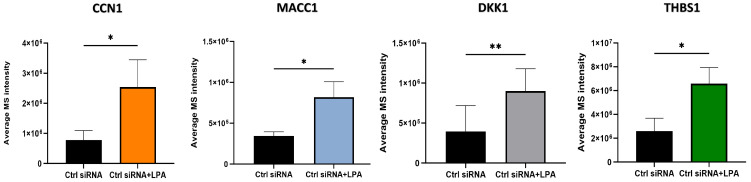
Quantified changes in expression levels of selected proteins up-regulated by LPA. Cells were treated for 3 h with and without 10 µM LPA for the proteomics analysis. Each bar represents mean ± SD from triplicate cell incubations; *, *p* < 0.05; **, *p* < 0.01.

Quantified proteomic results for proteins of particular interest are shown in Figure 2. Several of the proteins that were up-regulated by LPA, including CCN1, play cellular roles that are potentially relevant to the effects of LPA on prostate cancer cell adhesion and migration. MACC1 is an oncogene product that serves as a prognostic biomarker for multiple cancers [20]. MACC1 prominently enhances tumor cell migration and also inhibits immune surveillance. While the precise mechanisms are still being elucidated, MACC1 exerts multiple signaling effects in tumor cells including Erk and Akt activation. MACC1 effects on cell–ECM interactions are complex and may vary between cell types. MACC1 protein levels are regulated both transcriptionally and post-transcriptionally; the latter involving epigenetic regulation as well as protein stabilization [21]. Thrombospondin-1 (TSP1), like CCN1, is a matricellular protein that interacts with cell surface receptors as well as extracellular matrix components [22]. TSP1 has many physiological roles, including in wound healing, but is not generally overexpressed in tumor cells. Nonetheless, TSP1 contributes to cell adhesion and migration, and suppresses immune responses [23]. JUNB is a transcription factor that is one component of AP-1 complexes [24]. It is up-regulated during the S-phase of the cell cycle, and is important for cell division, but also can play roles in epithelial–mesenchymal transition and tumor cell invasion. Previous work has identified a role for JUNB in CCN1-mediated responses [25]. DKK1 is a secreted protein that is a prognostic biomarker for multiple cancers, including prostate cancer [26]. DKK1 modulates immune cell function in a way that contributes to an immunosuppressive tumor microenvironment and affects cell adhesion and motility to enhance metastasis. Together, the up-regulation of CCN1, MACC1, TSP1, JUNB, and DKK1 point to multiple steps by which LPA can potentially regulate cell–ECM interactions.

Interestingly, our analysis of down-regulated proteins (Table 2) also showed lowered levels of the LPA receptor, LPAR1, which mediates most of the effects of LPA in PC-3 cells [4]. A decrease in LPAR1 could be anticipated, as GPCRs are frequently down-regulated following prolonged stimulation by agonists. Another protein down-regulated in response to LPA was thioredoxin interacting protein (TXNIP), which will be discussed in more detail below.

**Figure 3:**
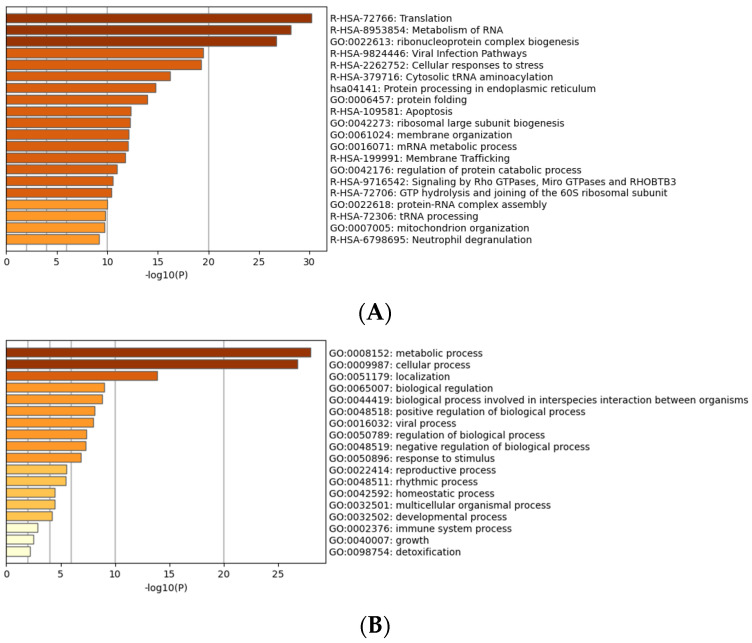
Gene ontology (GO) analysis of proteins differentially expressed in response to LPA. (**A**) Proteins grouped by molecular functions. (**B**) Proteins grouped by biological processes.

Gene enrichment analysis [27] was carried out to reveal major pathways impacted by LPA after 3 h. This analysis revealed that the up-regulated pathways included integrin signaling, mitosis, and wounding response, as well as negative regulation of cell junctions (Figure 3). All these pathways are consistent with known actions of LPA [5].

**Figure 4:**
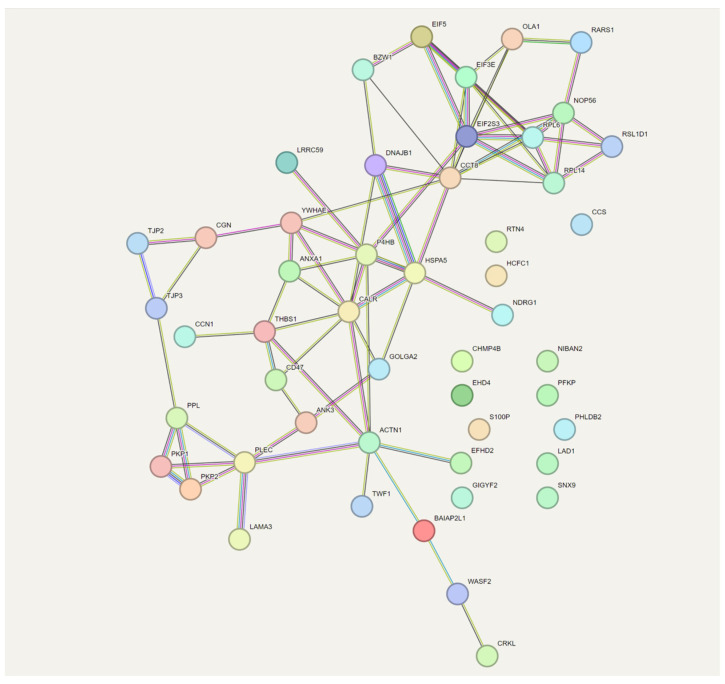
STRING analysis of proteins up-regulated by LPA that are related to cell adhesion. STRING analysis depicts protein-protein interaction networks. Different colored lines between proteins indicate various types of evidence (co-expression, experimental, database, etc.). The colors for each protein reflect their assignment to various protein families.

A STRING analysis (known and predicted protein–protein interaction analysis) was conducted to examine the interrelationships between proteins up-regulated by LPA that are related to cell adhesion (Figure 4). In this analysis, TSP-1 was identified as essential for linking CCN1 functionally to other LPA-responsive proteins.

### 2.2. Identification of Proteins Differentially Expressed after CCN1 Knockdown

The goal of this series of analyses was to determine whether knockdown of CCN1 would affect the proteomic responses observed in response to LPA. The cell incubations in which cells incubated with CCN1 siRNA were incubated with and without LPA were performed concomitantly with those discussed above in Section 2.1. Prior to proceeding with the proteomics analyses, successful knockdown of CCN1 was confirmed by immunoblotting of whole-cell extracts for CCN1. The conditions used for the CCN1 siRNA incubation had been optimized previously for a maximal decrease in CCN1 after LPA induction [17]; the extent of the knockdown was approximately 50%. Proteomics quantification and an immunoblot demonstrating the knockdown will be presented in Section 2.3. It should be noted that immunoblotting is inherently less quantitative than proteomics analysis.

**Figure 5:**
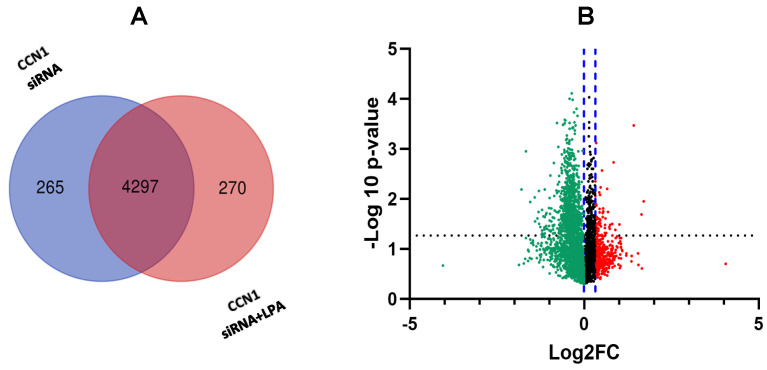
Proteomic differences observed in response to LPA after CCN1 knockdown. (**A**) Venn diagram showing number of proteins detected in the two treatment groups (cells incubated with and without LPA for three hours, after treatment with CCN1 siRNA). (**B**) Volcano plot showing proteins differentially expressed proteins between cells treated with and without LPA. FC = fold change (treated vs. untreated). The x-axis displays the fold-change; the y-axis shows statistical significance. The black dotted line indicates the threshold for statistical significance. The blue dotted lines indicate the region without statistically significant fold-change. Red and green colors indicate proteins up-regulated and down-regulated, respectively, in response to LPA.

As shown in Figure 5, 4297 proteins were detected in the CCN1 knockdown groups (± LPA), of which 276 proteins were uniquely expressed in cells treated with CCN1 siRNA and then incubated with LPA. From the overall analysis, many of the proteins that were most responsive to LPA before CCN1 knockdown were still up-regulated by LPA following CCN1 knockdown. However, our analysis revealed 30 LPA-responsive proteins for which the extent of the LPA induction was decreased after CCN1 expression was suppressed (Figure 1A). The differences in LPA response before and after CCN1 knockdown can be seen by comparing Table 1 and Table 3. In these two tables, CCN1, MACC1, DKK1, and TBS1 were all included as LPA-responsive proteins, but for each protein the fold increase in response to LPA was lower after incubation with CCN1 siRNA.

The proteins down-regulated by LPA (Table 4) were, in some respects, similar before and after CCN1 knockdown (compare Table 2 and Table 4). For example, LPAR1 was down-regulated before and after CCN1 expression was suppressed. Another protein that appears on both lists of down-regulated proteins (Table 2 and Table 4) is the thioredoxin interacting protein (TXNIP). TXNIP is a member of the arrestin family that plays complex biological roles, and can be either pro- or antitumorigenic in different types of cancers [28]. TXNIP has been reported to be decreased in prostate cancer and to act as a tumor suppressor; in one study, overexpression of TXNIP in PC-3 cells inhibited proliferation, migration, and invasion [29]. The role of TXNIP in LPA response in these cells had not been previously investigated, and deserves further attention since its LPA-induced down-regulation can potentially contribute to LPA-induced proliferation. There are also proteins in Table 4 that did not appear in Table 2, indicating that CCN1 knockdown enhanced the down-regulation of these proteins in response to LPA. These include FAM92B/ciBAR1, a protein that plays essential roles in ciliogenesis [30] and autophagy [31]. It should be noted that the serum starvation performed prior to our cell incubations, as a necessity to remove the LPA that is contained in serum, can induce a multitude of cellular responses [32] that include ciliogenesis and autophagy [33]. Thus, some of the proteomic responses to LPA likely reflect reversal of the effects of serum starvation. Another interesting protein that appeared on the list of proteins down-regulated by LPA only after CCN1 knockdown is syndecan-1 (SDC-1). CCN1 exerts many of its effects by binding to syndecan-4 [20]; however, both SDC-1 and -4 are involved in cell adhesion [34]. SDC-1 is overexpressed in cancers and is shed into the extracellular environment [35]. Our proteomic analysis of cell-associated proteins does not discern whether the LPA-induced decrease in SDC-1 might be due to enhanced shedding. Up-regulation of SDC-1 is associated with more aggressive prostate tumors [36].

The proteomic effects of CCN1 knockdown alone (without LPA) are also pertinent. A list of the top 20 proteins up- and down-regulated after CCN1 knockdown, without LPA treatment, is provided in the Supplementary Materials (Figures S1 and S2, Table S1). One interesting protein that is altered following CCN1 knockdown is prostate-associated microseminoprotein (MSMP/PSMP). The name “PSMP” refers to the fact that the protein was identified as being secreted by PC-3 cells [37]. Our results show that CCN1 knockdown *increased* levels of MSMP. The functions of MSMP have not been fully elucidated although it can act as a chemoattractant for macrophages [38]; the latter study indicated that MSMP is a chemokine ligand for the receptor CCR2. MSMP is up-regulated in prostate cancer cells in hypoxic conditions, resulting in increased drug resistance [39].

**Figure 6:**
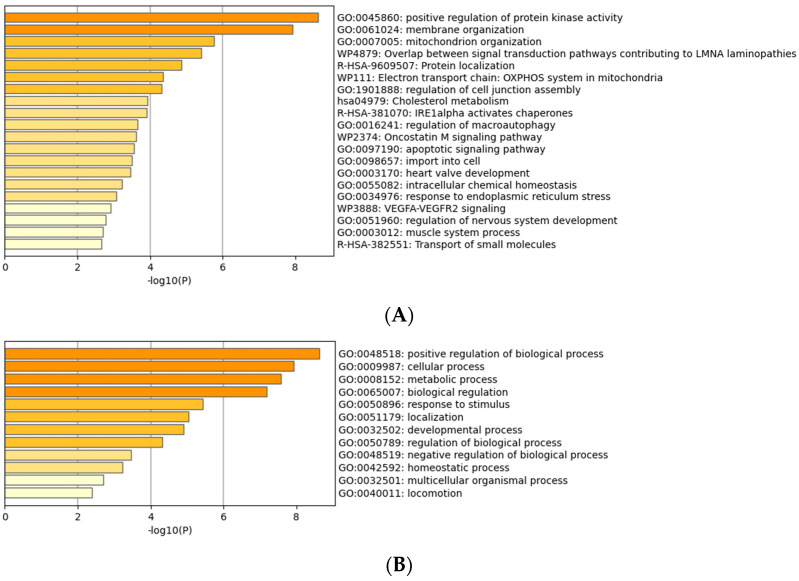
Gene ontology (GO) analysis of proteins differentially expressed in response to LPA after CCN1 knockdown. (**A**) Proteins grouped by molecular functions. (**B**) Proteins grouped by biological processes.

Gene ontology analysis of proteins differentially expressed in response to LPA after CCN1 knockdown is shown in Figure 6. The profile of the GO results is distinct from that observed before the reduction in CCN1 levels (compare Figure 3 and Figure 6). For example, involvement of the Rho pathway was on the list before CCN1 knockdown, but not after CCN1 knockdown. Positive regulation of protein kinase activity was prominent after CCN1 knockdown, but not before.

**Figure 7:**
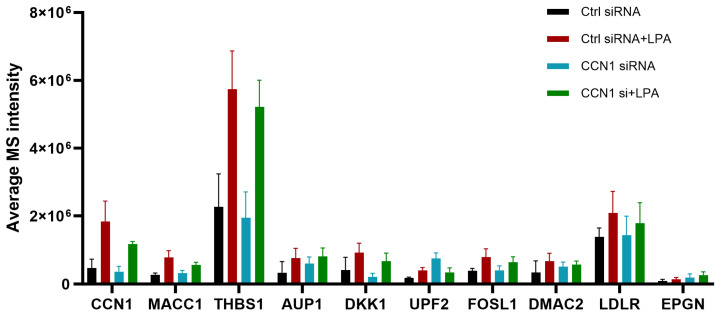
Proteins up-regulated in response to LPA and inhibited after CCN1 knockdown. The bar graph depicts average data independent acquisition values for selected top ten proteins where the LPA response (at 3 h) was inhibited after CCN1 knockdown.

The expression levels of LPA-responsive proteins in all four experimental conditions are presented in Figure 7. Notably, several of the proteins that were most responsive to LPA in the control situation were less responsive to LPA following CCN1 knockdown. These included proteins discussed earlier: CCN1, TSP1, MACC1, JUNB, and DKK1. Note that the net LPA response (not shown in Figure 7) is calculated by subtracting baseline expression, and that CCN1 knockdown alone affected basal expression in some cases.

### 2.3. Effects of CCN1 Knockdown on LPA-Induced Up-Regulation of MACC1 and TSP1

Based on the proteomics analyses, we selected MACC1 as an LPA-responsive protein worthy of further consideration. Our study showed that LPA induces MACC1 in PC-3 cells after three hours, and that CCN1 knockdown reduced the MACC1 protein levels (Table 1 and Table 3). These proteomic analysis results, presented as bar graphs in Figure 2 and Figure 7, confirm that the effect of LPA on MACC1 protein levels was decreased after CCN1 knockdown. The suppression of MACC1 induction was statistically significant but was partial, consistent with the partial knockdown of CCN1 that was achieved. To gain further insight into the changes in protein levels, we conducted immunoblotting experiments to examine the time course of the effects of LPA on MACC1, as well as the effects of CCN1 knockdown on this induction. Although immunoblotting results are less accurately quantifiable than a proteomics analysis, immunoblotting lends itself to rapid comparison of protein expression under multiple experimental conditions. 

**Figure 8:**
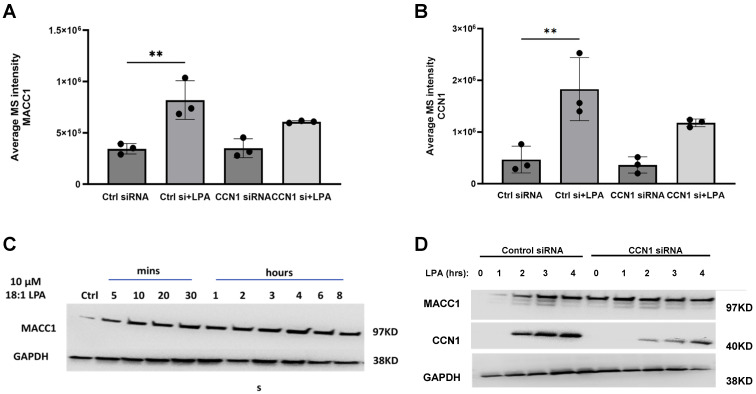
Effects of CCN1 knockdown on MACC1 and CCN1 1 protein Levels. (**A**,**B**) Expression levels of MACC1 (**A**) and CCN1 (**B**) were quantified from the proteomics data for cells incubated ± 10 µM LPA for 3 h, with and without CCN1 knockdown. These data are also presented in Figure 7. Data points represent mean ± SD of values from triplicate incubations; ** = *p* < 0.01. (**C**,**D**) Serum-starved PC-3 cells, incubated with control siRNA or CCN1 siRNA, were incubated with 10 µM LPA the indicated times. Whole-cell extracts were immunoblotted for MACC1, CCN1, and GAPDH (loading control) as indicated, on separate gels. Each blot is representative of results from three separate experiments.

The quantified effects of LPA on MACC1 and CCN1, before and after CCN1 knockdown, are shown in Figure 8A,B. Figure 8C presents the time course of the effects of LPA on MACC1 protein levels. The first observation is that the LPA-induced up-regulation of MACC1 is not as dramatic as that of CCN1. MACC1 is detected in PC-3 cells prior to LPA addition. In response to LPA, MACC1 protein levels increase to achieve maximal levels at 3–4 h, during the peak of CCN1 induction.

In Figure 8D, the time course of MACC1 up-regulation by LPA is compared between control cells and cells subjected to CCN1 knockdown. At the 3 h time point, MACC1 was visibly up-regulated by LPA in cells incubated with control siRNA, but not in cells incubated with CCN1 siRNA. As in Figure 8C, it is apparent that MACC1 is expressed in the absence of LPA, and that the extent of up-regulation is moderate. Interestingly, in both Figure 8C,D, LPA appears to increase the level of immunoreactive MACC1 at early times (5–60 min), prior to a transcriptional response. These results, which were not further explored here, suggest that LPA may alter the cellular distribution of MACC1, the retention of MACC1 in cells, and/or the accessibility of MACC1 to the immunoblotting antibody.

**Figure 9:**
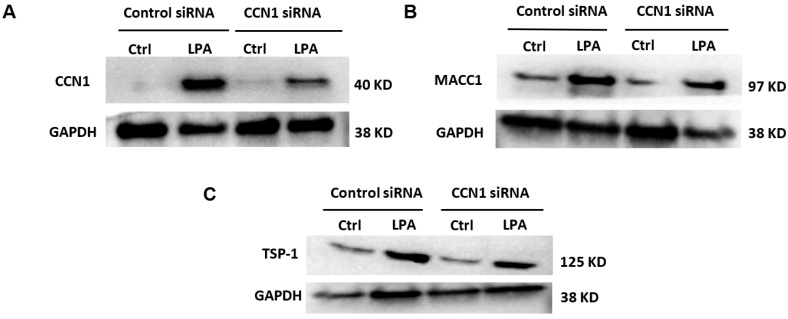
Effects of LPA on CCN1, MACC1, and TSP1 levels with and without CCN1 knockdown. (**A**–**C**): Serum-starved PC-3 cells that had been incubated with control siRNA or CCN1 siRNA were incubated for 3 h with 10 µM 18:1-LPA, with separate sets of cells for each panel. Whole-cell lysates were immunoblotted for CCN1 (**A**), MACC1 (**B**), TSP-1 (**C**), and GAPDH (loading control); the GAPDH blots were performed using the same samples but on separate gels.

In an additional series of immunoblotting experiments (Figure 9), the expression levels of CCN1, MACC1, and TSP1 were analyzed in cells incubated for three hours with and without LPA treatment, with and without CCN1 knockdown. TSP1 was re-examined because while it is clearly up-regulated by LPA (Figure 2), the effects of CCN1 knockdown on this response were more equivocal (Figure 7). The results of the immunoblots visually confirm the quantified results of the proteomics analysis, showing that LPA increases the levels of CCN1, MACC1, and TSP1, and that CCN1 knockdown partially inhibits the responses to LPA.

## 3. Discussion

This work examined events occurring in a human prostate cancer cell line three hours after the addition of LPA, a lipid growth factor that activates LPAR1 in these cells. Our study is the first to report the proteomic changes occurring in human prostate cancer cells after LPA treatment, and the first to examine the role of CCN1. Our observations corroborate our previous studies showing that LPA induces CCN1 in human prostate cancer cell lines [5,17]. The global proteomics analysis showed that CCN1 was one of the most highly up-regulated proteins in response to LPA.

Of particular importance, the results from this study show that a number of proteins related to cell adhesion, cell signaling, and the microenvironment are altered due to LPA treatment in PC-3 cells. The up-regulated proteins included TSP1, DKK1, JUNB, and MACC1. TXNIP was one of the proteins down-regulated by LPA. To our knowledge, the LPA-mediated modulations of these proteins (except for TSP1) are novel findings. The fact that expression levels were changed within three hours suggests that the identified proteins may play roles in bridging acute LPA-initiated signals with longer-term modulation of cellular function. While the analysis provided insight into a variety of proteins, it should be kept in mind that one of the limitations of this study is that all the proteomic differences were captured only at a 3 h time point, which may not represent the optimal time for all of the proteins. This unique time point was chosen to allow examination of the role of CCN1, which is induced by LPA after 2–4 h.

With respect to the role of CCN1, our results showed that CCN1 knockdown suppressed the LPA-induced up-regulation of multiple proteins in PC-3 cells, including ECM-related proteins. Consistent with these results, the STRING analysis identified adhesion-related pathways that were linked to CCN1 via TSP1. One of the pathways identified in the GO analysis is the Rho pathway. Rho GTPases are primary downstream components that transduce signals in response to LPA [6]. The GO profile for LPA response was quite different before and after CCN1 knockdown, particularly with respect to the Rho pathway which was prominent only in the presence of CCN1. This is interesting since Rho is acutely activated by LPA; alterations in Rho-related proteins seen three hours after LPA addition could reflect compensatory changes following Rho activation, but the results of the CCN1 knockdown suggest that the Rho pathway is also modulated by CCN1. More specifically, our results showed that RAC2, a member of RAC family of Rho GTPases involved in cell adhesion molecular function, was up-regulated by LPA in a manner that was suppressed by CCN1 knockdown. RACs function through regulating and organizing cytoskeleton, enhancing cell migration and cell spreading. This mechanism is usually regulated by the PAK4/LIMK1/Cofilin pathway, as demonstrated in CaP cells [40]. Our data also showed that Cofilin-2 was one of the LPA-responsive proteins decreased in response to CCN1 knockdown.

We chose to investigate MACC1 in more detail. LPA up-regulation of MACC1 was inhibited after CCN1 knockdown. Previous studies have shown a positive correlation between CCN1 and MACC1 in colorectal cancer [41]. MACC1 and CCN1 are both implicated in cancer metastasis, adhesion, and survival [21,42,43,44]. MACC1 was initially discovered in colorectal cancer [45]. It has been implicated in tumor progression and migration in various solid tumors, and is a potential prognostic marker in breast, colorectal, pancreatic, prostate, gastric and lung cancers [46,47,48,49]. A recent publication reports similar findings for prostate cancer [50]. While previous studies mention that overexpression of MACC1 is associated with poor prognosis in PC patients, the full spectrum of the cellular roles of MACC1 is still being determined. MACC1 regulates transcription of c-MET in the nucleus but is also found in cytoplasm and mitochondria [45]. MACC1 can participate in clathrin-mediated endocytosis and receptor recycling [51]. Thus, the roles and regulation of MACC1 in prostate cancer require further investigation.

MACC1 potentially participates in LPA-mediated signaling. In terms of cellular responses relevant to cancer, MACC1 has primarily been implicated in cell migration [52], an important event during tumor dissemination [53], but is also involved in other events including cell adhesion [21,54]. The complex process of cell migration results in cancer metastasis and is dependent on several events such as reorganization of cytoskeleton, and cell–matrix adhesion to facilitate anchorage points in the extracellular matrix space [55]. MACC1 is directly phosphorylated by MEK1, a component of the Erk mitogen-activated protein kinase (MAPK) pathway, resulting in enhanced Erk activation as well as migration and metastasis in colon cancer cells [56]. MACC1-enhanced transcriptional events contribute to epithelial–mesenchymal transition (EMT) in pancreatic cancer cells [57]. Previous work showed that CCN1 and MACC1 cooperate to promote human colorectal cancer cell metastasis, adhesion, migration, differentiation, angiogenesis, and survival [33]. In summary, emerging evidence suggests a potential interplay between MACC1 and CCN1.

While our results suggest that CCN1 plays a role in maintaining MACC1 levels in PC-3 cells, we have not tested the extent to which MACC1 is pre- and/or post-transcriptionally regulated by LPA in these cells. Although previous studies have reported the transcriptional induction of MACC1 by various receptor agonists [51,58], a detailed time course has usually not been examined. In addition, LPA has not been previously reported to increase MACC1 levels. These are novel aspects of the current study. The immunoblotting time course (Figure 8) shows that MACC1 is expressed in serum-starved cells prior to LPA addition. Although MACC1 levels are reproducibly increased 3 h after LPA addition, the extent of the increase ~2-fold) is relatively modest. Thus, LPA could potentially increase cellular MACC1 levels by stabilizing the protein (for example), rather than by increasing its transcription. Also, it is important to note that our whole-cell extracts included detergent-soluble cellular proteins, and not ECM proteins. Events altering protein secretion or solubility could potentially affect the proteomic results. With respect to possible early (<60 min) effects of LPA on MACC1 levels (Figure 8), MACC1 can be phosphorylated by MEK1 (the protein kinase that phosphorylates Erk) [56], and Erk is activated within 5 min of LPA addition to PC-3 cells [1,17]. Thus, both early and intermediate signaling events may impact MACC1 activity and protein levels through mechanisms that remain to be addressed.

CCN1 knockdown suppressed the ability of LPA to increase MACC1 levels. Again, several mechanisms could be in play. Although CCN1 is a secreted protein, it has been suggested to play roles in the nucleus [59,60]. Thus, CCN1 could conceivably alter protein expression at the transcriptional level, either directly or indirectly through integrin-mediated signaling events. On the other hand, CCN1 has also been shown to alter the levels of other proteins via protein–protein interactions [61]. In this way, CCN1 could either stabilize or destabilize its binding partners. Further exploration of the interactions between CCN1 and MACC1 are warranted.

Turning to other proteins up-regulated by LPA, thrombospondin-1 is a secreted protein that regulates cell adhesion by binding to integrins [22]. Its role in the ECM is complex and context-dependent, but, in general, TSP1 levels are lower in the tumor microenvironment, and overexpression of TSP1 inhibits tumor growth while enhancing invasion. However, tumor-promoting effects of TSP1 have also been reported. TSP-1 is an inducible protein, and its levels have been reported to increase in response to LPA in several model systems. For example, LPA induces TSP1 at the transcriptional level in rat cortical astrocytes through the LPA receptor LPAR1 [62]. LPA has been previously shown to induce TSP1 in PC-3 cells after an 8 h treatment [63]. Since both CCN1 and TSP1 bind to integrins, the net effect of simultaneously increasing both CCN1 and TSP1 is difficult to predict. It is intriguing that STRING analysis (Figure 4) identified a central role for TSP1. It is likely that, since TSP1 plays a negative role in cell–substrate adhesion, TSP1 up-regulation contributes to the transient nature of the effect of LPA on adhesion observed in our previous studies [17].

Other proteins of interest, identified in the proteomics analysis, are TXNIP and MSMP/PSMP. The roles of these proteins in prostate cancer cells in the context of LPA response remain to be fully explored.

Our results have paved the way towards exploring newer directions to understand the cellular roles of CCN1. This study has opened possibilities that deserve further exploration. Matricellular proteins such as CCN1 represent new targets for potential therapeutic interventions for prostate cancer, and in other diseases where CCN1 and/or the LPA-CCN axis play a crucial role.

## 4. Materials and Methods

### 4.1. Chemicals and Reagents

Dimethyl sulfoxide (DMSO), trypsin protease, iodoacetamide (IAA), and the bicinchoninic acid (BCA) protein assay kit were obtained from Thermo Scientific (Waltham, MA, USA). Sodium dodecyl sulfate (SDS) and bovine serum albumin (BSA) were purchased from Sigma Life Science (Burlington, MA, USA). Acetone and LC-MS-grade acetonitrile were purchased from Sigma Aldrich (St. Louis, MO, USA) and VWR BDH Chemical (Radnor, PA, USA), respectively. LC-MS-grade formic acid and methanol were procured from Fisher Chemical (Fair Lawn, NJ, USA). Dithiothreitol (DTT) was obtained from Fisher Bioreagents (Pittsburgh, PA, USA).

### 4.2. Cell Culture

PC-3 cells were obtained from the American Type Culture Collection (Manassas, VA, USA). The cells were grown in RPMI 1640 medium supplemented with 10% (*v*/*v*) fetal bovine serum (FBS) (Hyclone/Cytiva; Marlborough, MA, USA) and 50 U/mL penicillin/50 µg/mL streptomycin. The cells were maintained on standard tissue culture plastic in an incubator at 37 °C with 5% CO_2_.

### 4.3. Cell Incubations and Sample Preparation

PC-3 cells were seeded at 200,000 cells/well in a 6-well plate in RPMI-1640 media supplemented with 10% FBS. CCN1 siRNA complex, scrambled siRNA complex, and transfection medium were then incubated with the cells according to the manufacturer’s recommendations (Santa Cruz Biotechnologies; Santa Cruz, CA, USA) in a volume of 1 mL per well. The cells were incubated at 37 °C in a cell culture incubator for 5–7 h, then an additional 1 mL of RPMI medium supplemented with 20% FBS was added. After 18–24 h, the medium was changed to RPMI-1640 supplemented with 10% FBS. Cells were incubated for 48 h in a cell culture incubator, serum-starved for 24 h, then incubated with and without 10 µM LPA for 3 h. After removal of the media, the cells were washed thrice with ice cold PBS, and cell lysates were collected at 4 °C in an 8 M urea buffer (pH 8.0) containing 0.1 M ammonium bicarbonate and protease inhibitors. The lysates were subjected to BCA analysis to determine protein concentration. Following the above step, the lysate samples were incubated with 25 mM dithiothreitol (DTT) in deionized water at 37 °C for one hour. The sample was then alkylated with 60 mM IAA in dark and RT for 30 min. After adding sufficient 0.1 M ammonium bicarbonate to dilute the sample mixture to 1.6 M urea, the mixture was digested with trypsin overnight with a 1:40 (enzyme: protein) ratio at room temperature. The digested sample was desalted by passage through a C18 SPE cartridge (Waters Corporation, Pleasanton, CA, USA) and then dried down for LC/MS.

### 4.4. LC-MS Analysis

The proteomics data were acquired by analyzing the digested samples using Easy-nLC 200 coupled with a Q Exactive HF Hybrid Quadrupole-Orbitrap Mass Spectrometer (Thermo Scientific, Waltham, MA, USA). Peptides were separated using a Pepmap RSLC C18 (25 cm × 75 µm) column (2 µm, 100 A) with an 80 min gradient program (buffer A: 0.1% formic acid; buffer B: 80% acetonitrile with 0.1% formic acid) as follows: 0–5 min (2–6% buffer B), 5–60 min (6–30% buffer B), 60–65 min (30–100% buffer B), and 65–80 min (100% buffer B). The flow rate was 300 nL/min. MS analysis was performed with spray voltage of 1.7 kV in data-independent acquisition (DIA) mode. Global proteomics data were acquired using the following conditions: mass range (*m*/*z*), 350–1025 with variable isolation windows; MS1 resolution, 120,000; MS2 resolution: 30,000.

### 4.5. Immunoblotting

Whole-cell extracts containing equal amounts of protein (30 µg), prepared as described previously [17], were separated by SDS-PAGE on 12% or 15% Laemmli gels, transferred to PVDF membranes, and incubated with primary antibody (overnight at 4 °C) and then secondary antibody (1–2 h at room temperature) in Tris-buffered saline (pH 7.8) containing 4 μg/mL BSA and 0.1% Tween-20. Antibodies recognizing CCN1 and CCN2 were from Cell Signaling Technology (Danvers, MA, USA) (#14479S; #86641S). Antibodies recognizing MACC1 (#86290) and GAPDH were from Cell Signaling Technologies and Santa Cruz Biotechnologies, respectively. Secondary antibodies, anti-rabbit, and anti-mouse (IgG HRP-linked; #7076; #7076S, respectively) were obtained from Cell Signaling Technologies. Primary and secondary antibodies were used at 1:1000 and 1:2000 dilutions, respectively. Blots were developed using enhanced chemiluminescence reagents (Pierce Biotechnology, Thermo Fisher Scientific, Waltham, MA, USA) and imaged using a GelDoc system (Bio-Rad; Hercules, CA, USA). Protein expression was quantified by densitometry using ImageJ software (https://imagej.net/ij/ access date 12 December 2023). Results were normalized to the GAPDH loading control after background subtraction.

### 4.6. Data Analysis

LC-MS data were searched using DIA-NN (version 18.1.1) (https://github.com/vdemichev/DiaNN; access date 1 December 2023), with library-free analysis. Deep learning-based in silico spectral library generation was enabled with human FASTA database. The maximum number of missed cleavages was 1 as default. Fixed medication includes carbamidomethylation, N-term M excision, and variable modification only sets oxidation (M). Unrelated run and MBR (match between run) was selected, and all other parameters used the default setting.

Proteins were identified with 1% false discovery rate (FDR). Further processing of data was performed using Microsoft Excel (Microsoft 365 MSO version 2401) and Graph Pad PRISM (Prism 10 for Windows 64-bit).

Proteins that were found in 2 out of 3 replicates were considered detectable. Proteins found in 1 out of 3 replicates were considered not detected. All detected proteins were assessed for the total protein count, which was visualized by Venn diagram (http://bioinformatics.psb.ugent.be/webtools/Venn/; access date 9 December 2023). Proteins detected in 2 out of 3 replicates were compared for their abundance using volcano plots where the minimum log fold change was 1.25 and the significance value was <0.05. Pathways were determined by utilizing the STRING database (www.string-db.org; access date 14 December 2023) and Metascape (www.metascape.org; access date 10 December 2023).

## Figures and Tables

**Table 1:** Proteins significantly up-regulated in response to LPA. The top twenty proteins with respect to fold change (with LPA ÷ without LPA) are listed.

Protein Name	Description	Fold Change
CCN1	CCN family member 1	3.9
MACC1	Metastasis-associated in colon cancer protein 1	2.8
THBS1	Thrombospondin-1	2.5
DKK1	Dickkopf-related protein 1	2.3
UPF2	Regulator of nonsense transcripts 2	2.2
FOSL1	Fos-related antigen 1	2.0
SEC22A	Vesicle-trafficking protein SEC22a	2.0
LRCH4	Leucine-rich repeat and calponin homology domain-containing protein 4	2.0
DMAC2	Distal membrane-arm assembly complex protein 2	2.0
EFR3A	Protein EFR3 homolog A	1.7
CELSR1	Cadherin EGF LAG seven-pass G-type receptor 1	1.7
TMEM167A	Protein kish-A	1.7
MAGT1	Magnesium transporter protein 1	1.7
JUNB	Transcription factor jun-B	1.6
ERMP1	Endoplasmic reticulum metallopeptidase 1	1.5
OSTC	Oligosaccharyltransferase complex subunit OSTC	1.5
LDLR	Low-density lipoprotein receptor	1.5
KRR1	KRR1 small subunit processome component homolog	1.5
PWP1	Periodic tryptophan protein 1 homolog	1.5
CYB5R1	NADH-cytochrome b5 reductase 1	1.5

**Table 2:** Proteins significantly down-regulated in response to LPA. The top ten proteins with respect to fold change (with LPA ÷ without LPA) are listed.

Protein Name	Description	Fold Change
LPAR1	Lysophosphatidic acid receptor 1	0.22
PTMS	Parathymosin	0.24
TXNIP	Thioredoxin-interacting protein	0.30
PGM3	Phosphoacetylglucosamine mutase	0.35
DYNLT3	Dynein light chain Tctex-type 3	0.36
SENP1	Sentrin-specific protease 1	0.36
VOPP1	WW domain binding protein VOPP1	0.37
RASA1	Ras GTPase-activating protein 1	0.41
PPID	Peptidyl-prolyl cis-trans isomerase D	0.43
ILK	Integrin-linked protein kinase	0.48

**Table 3:** Proteins significantly up-regulated by LPA in cells incubated with CCN1 siRNA. The top twenty proteins with respect to fold change (with LPA ÷ without LPA) are listed.

Protein Name	Protein Description	Fold Change
CCN1	Cysteine rich angiogenic factor-61	3.25
DKK1	Dickkopf-related protein 1	3.11
THBS1	Thrombospondin-1	2.68
MAN1A2	Alpha-mannosidase 2	2.01
MT-ND1	NADH-ubiquinone oxidoreductase chain 1	1.86
SORT1	Sortilin	1.79
MACC1	Metastasis-associated in colon cancer protein 1	1.75
NDC1	Nucleoporin NDC1	1.74
TOR1B	Torsin-1B	1.67
ALG5	Dolichyl-phosphate beta-glucosyltransferase	1.65
TWISTNB	DNA-directed RNA polymerase I subunit RPA43	1.64
ERN1	Serine/threonine-protein kinase/endoribonuclease IRE1	1.61
FOSL1	Fos-related antigen 1	1.58
OSBPL8	Oxysterol-binding protein-related protein 8	1.54
VMA21	Vacuolar ATPase assembly integral membrane protein VMA21	1.53
CCDC71L	Coiled-coil domain-containing protein 71L	1.52
COL6A2	Collagen alpha-2(VI) chain	1.51
CDKN1A	BRCA2 and CDKN1A-interacting protein	1.50
HIP1R	Huntingtin-interacting protein 1-related protein	1.50
TMEM254	Phospholipid transfer protein C2CD2L	1.47

**Table 4:** Proteins significantly down-regulated by LPA in cells incubated with CCN1 siRNA. The top ten proteins with respect to fold change (with LPA ÷ without LPA) are listed.

Protein Name	Description	Fold Change
TXNIP	Thioredoxin-interacting protein	0.29
LPAR1	Lysophosphatidic acid receptor 1	0.31
FAM92B	CBY1-interacting BAR domain-containing protein 2	0.34
HSF1	Heat shock factor protein 1	0.37
ALAS1	5-aminolevulinate synthase, nonspecific, mitochondrial	0.38
GTPBP1	GTP-binding protein 1	0.39
PRKAG1	5′-AMP-activated protein kinase subunit gamma-1	0.40
SDC1	Syndecan-1	0.41
KYNU	Kynureninase	0.41
HMBS	Porphobilinogen deaminase	0.42

## Data Availability

The data presented in this study are available on request from the corresponding author. The proteomics data are in the process of being archived in the PRIDE Proteomics Identifications database.

## Supplementary Materials

The supporting information can be downloaded at: https://www.mdpi.com/article/10.3390/ijms25042067/s1.
