# Supplementary material for: Global Proteomics Analysis of Lysophosphatidic Acid Signaling in PC-3 Human Prostate Cancer Cells: Role of CCN1
Source: Int J Mol Sci. 2024 Feb 8;25(4):2067. doi: 10.3390/ijms25042067 (PMC10889543; doi:10.3390/ijms25042067)
Supplement: Supplementary file 1 [file ijms-25-02067-s001.zip › ijms-2829195-supplementary.pdf]

Supplementary Figure S1.

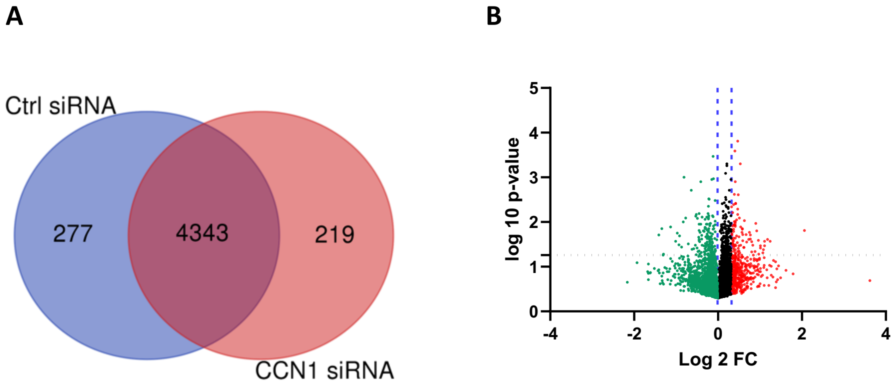

Supplementary Figure S1. Proteomic differences observed after CCN1 knockdown. *Panel A*: Venn diagram showing number of proteins detected in the two treatment groups (cells incubated with and without CCN1 siRNA). *Panel B:* Volcano plot showing proteins differentially expressed proteins between cells treated with and without CCN1 siRNA. FC = fold change (treated vs. untreated). Red and green colors indicate proteins up-regulated and down-regulated, respectively, in response to CCN1 knockdown.

Supplementary Table S1

Supplementary Table S1. Proteins significantly up- or down-regulated in cells incubated with CCN1 siRNA.

The top ten proteins with respect to fold change (with CCN1knockdown/without CCN1 knockdown) are listed.

Genes	Description	Fold Change
UPF2	Regulator of nonsense transcripts 2	2.24
AVL9	Late secretory pathway protein AVL9 homolog	1.04
MAP3K20	Serine/threonine-protein kinase Chk2	1.4E-06
MMAB	Corrinoid adenosyltransferase MMAB	1.71
SUN2	SUN domain-containing protein 2	1.56
ITPA	Inosine triphosphate pyrophosphatase	0.57
FRMD8	FERM domain-containing protein 8	1.30
RNF13	E3 ubiquitin-protein ligase RNF13	1.02
BCR	Breakpoint cluster region protein	1.48
MCU	Calcium uniporter protein, mitochondrial	1.91
EFR3A	Protein EFR3 homolog A	1.75
TUBB3	Tubulin beta-3 chain	1.28
PEX16	Peroxisomal membrane protein PEX16	1.51
TMEM41B	Transmembrane protein 41B	1.75
AUP1	Lipid droplet-regulating VLDL assembly factor AUP1	2.26
GEMIN4	Gem-associated protein 4	1.34
FNTA	Protein farnesyltransferase/geranylgeranyltransferase type-1 subunit alpha	0.56
MSMP	Prostate-associated microseminoprotein	1.56
RPS27A	Ubiquitin-ribosomal protein eS31 fusion protein	1.40
CLDN4	Claudin-4	1.78

Supplementary Figure S2.

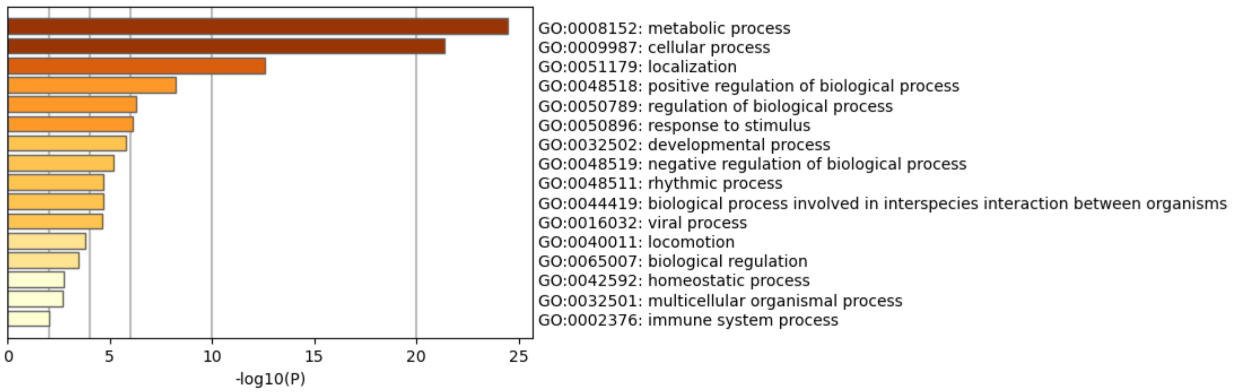

Supplementary Figure S2: Gene ontology (GO) analysis of proteins differentially expressed in response to CCN1 knockdown. Proteins were grouped by biological processes.
